# Identification and verification of immune-related biomarkers and immune infiltration in diabetic heart failure

**DOI:** 10.3389/fcvm.2022.931066

**Published:** 2022-11-17

**Authors:** Zuoquan Zhong, Hanlin Zhang, Ting Xu, Jinjin Hao, Xing Chen, Shimin Sun, Jinjin Yang, Jing Sun, Hui Lin, Hangyuan Guo

**Affiliations:** ^1^Department of Cardiology, Shaoxing People’s Hospital, Shaoxing Hospital of Zhejiang University, Shaoxing, Zhejiang, China; ^2^The First Clinical Medical College, Wenzhou Medical University, Wenzhou, Zhejiang, China; ^3^Department of Cardiology, Zhejiang University School of Medicine, Hangzhou, Zhejiang, China; ^4^Department of Respiratory Medicine, Shaoxing People’s Hospital, Shaoxing Hospital of Zhejiang University, Shaoxing, Zhejiang, China; ^5^Department of Cardiovascular, Lihuili Hospital Affiliated to Ningbo University, Ningbo, Zhejiang, China; ^6^College of Medicine, Shaoxing University, Shaoxing, Zhejiang, China; ^7^Shaoxing People’s Hospital, Shaoxing Key Laboratory of Cardio-Cerebral Vascular Disease Rehabilitation Technology Research, Shaoxing, Zhejiang, China

**Keywords:** diabetic heart failure, diabetic cardiomyopathy, immunomodulation, bioinformatics, cardiac function

## Abstract

**Purpose:**

Diabetic heart failure (DHF) or cardiomyopathy is a common complication of diabetes; however, the underlying mechanism is not clear. In the present study, the authors searched for differentially expressed genes associated with DHF and the molecular types of immune cells based on bioinformatics.

**Methods:**

The RNA expression dataset of DHF was obtained from the NCBI Gene Expression Omnibus (GEO) database. After preprocessing the data, the differentially expressed genes (DEGs) between the DHF group and the non-diabetic heart failure (NHF) group were screened and intersected with immune-related genes (IRGs) in the ImmPort database. Gene Ontology (GO) and Kyoto Encyclopedia of Genes and Genomes (KEGG) enrichment analyses were performed using the DAVID tool. The ssGSEA algorithm was used to evaluate immune infiltration of the heart tissue in each group. In addition, the protein-protein interaction (PPI) network and miRNA-mRNA network were constructed using the STRING online website and Cytoscape program. Finally, validation analysis was performed using animal models.

**Results:**

Eight immune-related core genes were identified. GO and KEGG showed that core genes were mainly enriched in angiogenesis and cytokine-cytokine receptor interaction. Immune infiltration results showed that activated dendritic cells, central memory CD4 T cells, central memory CD8 T cells, myeloid-derived suppressor cells (MDSCs), neutrophils, and regulatory T cells may be involved in DHF. Neutrophils may play a key role in the pathogenesis of HF in diabetes.

**Conclusion:**

Immune-related core genes and immune infiltrating cells provide a new perspective on the pathogenesis of DHF.

## Introduction

Heart failure (HF) is the final stage of heart disease and is a great burden on individuals and society. The prevalence of HF is also increasing annually. It has been estimated that the prevalence of HF in the United States will increase from 2.42% in 2012 to 2.97% in 2030 (1). Although the mechanism of HF is complex, persistent inflammation and immune damage dominate it. As a conventional cytokine, tumor necrosis factor (TNF) is rarely expressed in the normal myocardium, but is highly expressed in the pathological heart and causes cardiomyocyte apoptosis (2). In myocardial ischemia, interleukin (IL)-6 is produced in large quantities, resulting in cardiomyocyte hypertrophy and apoptosis (3). Recent studies have shown that cardiac lesions are related to immune cell infiltration (4, 5). The interaction between various immune cells and cytokines leads to injury of the terminal cardiovascular system.

Diabetes is a serious public health problem and an independent risk factor for HF. In a large-scale clinical trial, patients with HF with diabetes had more adverse factors than patients with HF without diabetes, and the probability of cardiovascular death, hospitalization for HF, or urgent HF visit significantly increased (6). Studies have shown that patients with diabetes are more prone to vascular endothelial cell dysfunction (7) and coronary microvascular injury. Although many drugs, such as insulin, sulfonylureas, and thiazolidinediones, have good hypoglycemic effects, long-term use aggravates HF (8). Therefore, it is necessary to understand the pathogenesis of HF in patients with diabetes. However, the relationship between diabetic heart failure (DHF) and immune cell infiltration remains unclear.

In recent years, with the rapid development of microarray and high-throughput sequencing technology, pathogenic genes and core pathways of diseases have been effectively identified through bioinformatics analysis. In this study, the NCBI Gene Expression Omnibus (GEO) database was used to identify differentially expressed genes (DEGs) in DHF and non-diabetic heart failure (NHF) and core genes were obtained from interrelated immune genes. Gene Ontology (GO) and Kyoto Encyclopedia of Genes and Genomes (KEGG) enrichment analyses, immune cell infiltration analysis, and miRNA-mRNA network construction were then conducted. Finally, the expression of core genes was verified using animal models.

## Materials and methods

### Data collection

The mRNA expression dataset GSE26887 (Affymetrix Human Gene 1.0 ST Array) was obtained from the NCBI GEO.^1^ The GSE26887 dataset contained 19 heart samples, including 7 from patients with DHF and 12 from patients with NHF (9). Cardiac biopsy is mainly derived from the left ventricle in the non-infarcted area. HF patients were matched on most characteristics, like age, sex, ethnic distribution to reduce experimental error. Raw CEL files were obtained, and raw data were processed using the “affy” package. The “limma” package was used to identify DEGs between the two groups. The volcano map and heatmap were drawn by the “ggplot2” package for data visualization. Thresholds of DEGs were |log2 (fold change)| > 0.05 and *p* < 0.05. The immune-related genes (IRGs) were obtained from the ImmPort database.^2^ The overlapping genes (DE-IRGs) of the DEGs and IRGs were used for further analysis.

### Functional enrichment analysis and immune infiltration

The Database for Annotation, Visualization, and Integrated Discovery (DAVID) (10) was used for GO and KEGG enrichment analyses of DE-IRGs. To investigate the infiltration of 28 immune cells in DHF and NHF heart samples, ssGSEA using the R package “GSVA” was performed. The ssGSEA method allowed the definition of an enrichment fraction that represented the absolute enrichment level of each gene set in each dataset. The gene expression value of given sample was normalized by rank, and the enrichment score was generated using the empirical cumulative distribution function of the genes in the feature and the remaining genes (11, 12). According to the enrichment score, analyze the infiltration level of immune cells in myocardial tissue. Spearman correlation analysis was used to explore the correlation between immune cells and core genes with the help of the “corrplot” R package.

### Protein-protein interaction network

DE-IRGs were imported into the STRING database^3^ to analyze protein-protein interactions (PPIs), and the minimum required interaction score was 0.4. The results were reconstructed using Cytoscape software. The MCC algorithm of cytohubba was used to screen for core genes.

### Prediction of miRNA-mRNA interactions

The miRDB^4^ (13) and miRTarBase (14)^5^ databases were used to predict mRNA-targeted miRNAs. The intersections of mRNA-targeted miRNAs from the two databases were used to construct the mRNA-miRNA network.

### Validation of core genes

The area under the receiver operating curve (ROC) was used to assess the ability of the core gene to predict DHF. Sixteen male 6-week-old C57BL/BJ mice, eight male 6-week-old db/db mice and eight male 6-week-old db/m mice were purchased from Nanjing Biomedical Research Institute of Nanjing University (China). All mice were grown in a specific-pathogen-free (SPF)-grade environment. All procedures complied with the standards of the Animal Ethics Committee. In order to build type 1 diabetes mice, Sixteen mice were randomly divided into a DHF group (*n* = 8) and a control group (*n* = 8) after 1 week of adaptive feeding. The DHF group received a high-fat and high-sugar diet for 4 weeks, and intraperitoneal injection of streptozotocin (STZ; 20 mg/kg) was started from the fifth week to the eighth week. Thereafter, the mice were fed a high-fat and high-sugar diet for 20 weeks. The control group received the same amount of normal saline and a normal diet. In addition, in order to construct type 2 diabetes mice, eight db/db mice were fed a high-fat diet and db/m mice were fed the same amount of normal water and food. After 4 weeks, mice with fasting blood glucose ≥ 11.1 mmol/L were regard as successful type 2 diabetes mice.

### Echocardiography

After 20 weeks, the mice were anesthetized through intraperitoneal injection of pentobarbital, and left ventricular end-diastolic dimension (LVEDD), left ventricular end-systolic dimension (LVESD), left ventricular ejection fraction (LVEF), and left ventricular systolic fraction (LVFS) were detected using a small animal ultrasonic probe.

### Tissue sample collection and reverse transcription-polymerase chain reaction detection

**FIGURE 1:**
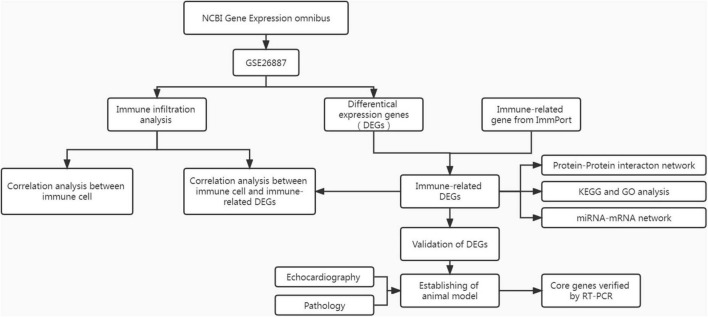
The flowchart of analysis process.

The heart tissues of the mice were fixed in paraformaldehyde and used for paraffin sections. The hearts were stained with hematoxylin and eosin (H&E) and Masson’s trichrome stain. Other mouse heart tissues were collected to prepare the homogenate. Total RNA was extracted from homogenates using a commercial kit (ES Science, China) and miScript II RT Kit (QIAGEN, Germany). All RNA reverse-transcribed into cDNA. Reverse transcription-polymerase chain reaction (RT-PCR) was performed using a PCR instrument (Roche, Switzerland). β-Actin and U6 were used as internal parameters. The relative mRNA and miRNA expression were computed using the 2^–ΔΔCt^ method. Primer sequences for each mRNA and miRNA are displayed in Supplementary Table 1. A flow chart of this study is shown in Figure 1.

### Statistical analysis

Data are presented as the mean ± standard deviation (*SD*). Statistical significance was set at *P* < 0.05. All statistical analyses were performed using R software (version 4.1.0) and GraphPad Prism 8.

## Results

### Identification of differentially expressed genes

**FIGURE 2:**
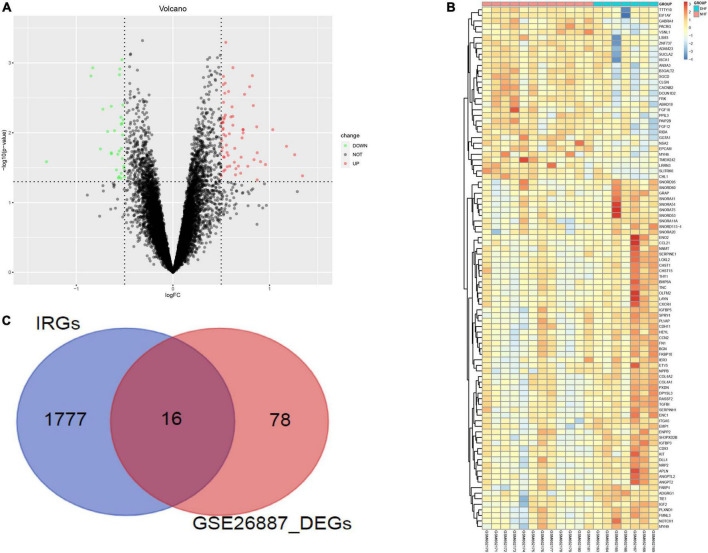
Identification of differential genes related to immunity. **(A)** DEGs between DHF patients and NHF patients (Red, up-regulated in DHF; green, down-regulated in NHF). **(B)** Heatmap of 94 DEGs. **(C)** Intersection of DEGs and immune related genes.

**FIGURE 3:**
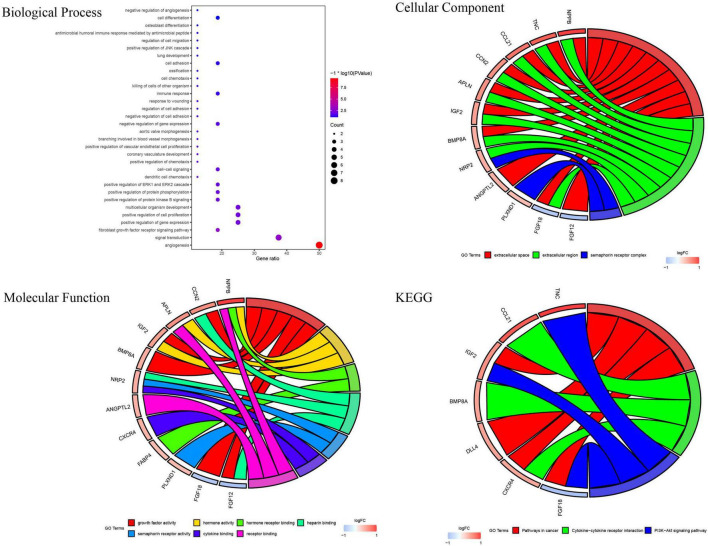
GO and KEGG enrichment analysis of immune related DEGs.

GSE26887 is a dataset of patients with post-ischemic HF. Myocardial biopsies were obtained from non-ischemic remote areas of the left ventricle in seven patients with DHF and 12 patients with NHF. A total of 94 DEGs were identified in the myocardial tissue of patients with DHF compared with NHF myocardial tissue, including 63 upregulated and 31 downregulated genes (Figures 2A,B). GO and KEGG enrichment analyses were performed to explore the biological functions of the DEGs. As shown in Supplementary Figure 1, the DEGs were highly related to angiogenesis, extracellular matrix organization, integrin binding, and the phosphatidylinositol 3-kinase (PI3K)/protein kinase B signaling pathway. Next, we used the intersection of DEG and IRGs and performed enrichment analysis on DE-IRGs (Figure 2C). GO analysis suggested that DE-IRGs were enriched in angiogenesis, signal transduction, and fibroblast growth factor receptor (FGFR) signaling factor in the biological process, and they were associated with cytokine binding, growth factor activity, and hormone activity in molecular function. KEGG results indicated that DE-IRGs were enriched in the PI3K-Akt signaling pathway, cytokine-cytokine receptor interaction, and pathways in cancer (Figure 3).

### Protein-protein interaction network construction and screening of core genes

**FIGURE 4:**
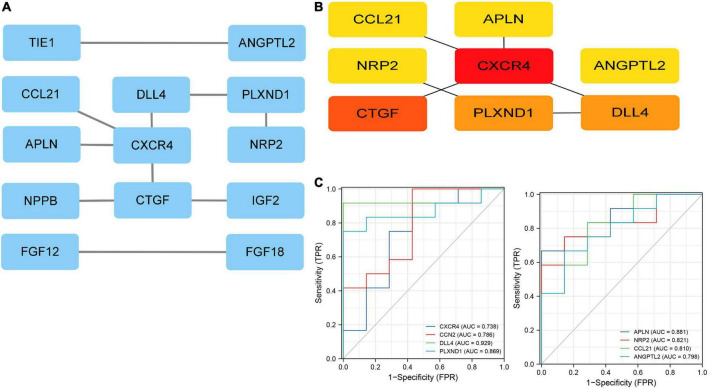
Protein-protein interaction (PPI) network. **(A)** The PPI network of 19 immune related DEGs. **(B)** The top eight genes with the highest connection. **(C)** ROC curves for eight immune related genes. ROC, receiver operating characteristic.

The DE-IRGs were imported into the STRING database (see the text footnote 3). The core genes were identified using the Cytohubba plugin. Finally, eight core genes with the highest scores were selected using the MCC algorithm: C-X-C motif chemokine receptor 4 (CXCR4), cellular communication network factor 2 (CCN2), delta-like canonical Notch ligand (DLL4), plexin D1(PLXND1), apelin (APLN), neuropilin 2 (NRP2), C-C motif chemokine ligand 21 (CCL21), and angiopoietin-like 2 (ANGPTL2) (Figures 4A,B).

### Immune-infiltration analysis

**FIGURE 5:**
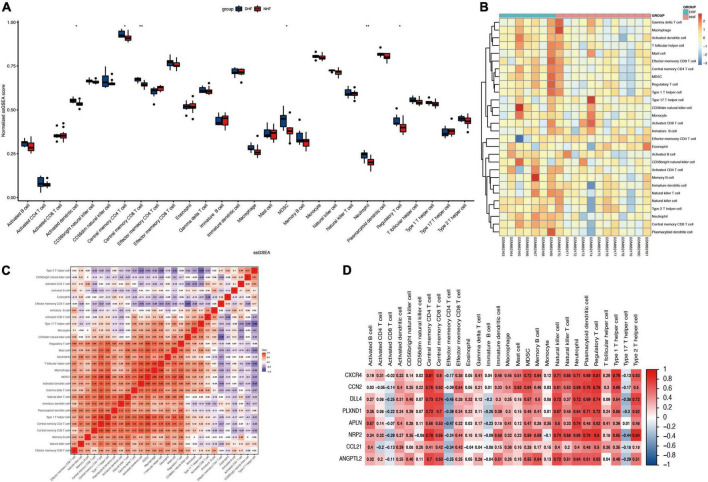
Immune infiltration in myocardial fissure. **(A)** Relative ssGSEA score of 28 immune cells. **(B)** Distribution of immune cells in each sample. **(C)** Correlation analysis between immune infiltrating cells. **(D)** Correlation analysis between immune infiltrating cells and immune related genes. *P < 0.05; **P < 0.01.

Immune infiltration of heart samples from patients with DHF or NHF was evaluated using the ssGSEA algorithm, and the immune scores were normalized to show the results more intuitively. As shown in Figure 5A, the scores of activated dendritic cells, central memory CD4 T cells, central memory CD8 T cells, myeloid-derived suppressor cells (MDSCs), neutrophils, and regulatory T cells were higher in the DHF heart sample than in the NHF sample. In addition, as illustrated in the heatmap (Figure 5B), there were significant differences in immune-infiltrating cells between the DHF and NHF heart samples. Additionally, an immune cell correlation network was constructed (Figure 5C). There was a high positive correlation between the immune cells with the above significant difference scores, and type 2 helper cells correlated negatively with type 17 helper cells (–0.66).

### Association of core genes with immune cells

**FIGURE 6:**
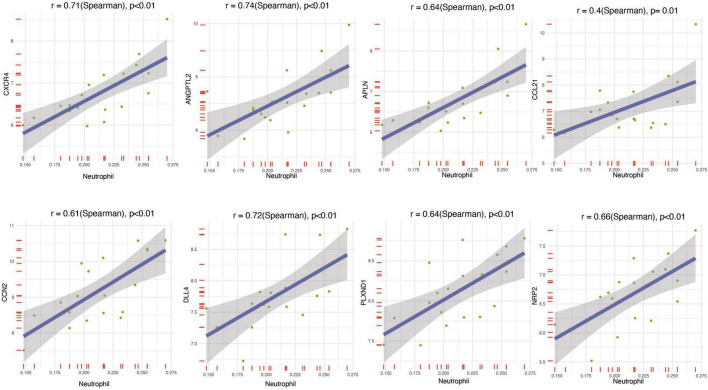
Correlation analysis between immune infiltrating cells and neutrophils.

To understand the relationship between the core genes and immune cells, a correlation analysis was conducted. The results indicated that neutrophils were positively correlated with core gene expression (Figure 5D). Therefore, the correlations between neutrophils and core genes were individually analyzed and the result confirmed the positive correlation (Figure 6). The correlation between core genes and neutrophils was greater than 0.4. In particular, the correlation coefficients of CXCR4, ANGPTL2, and DLL4 were greater than 0.7, and the *P*-values were less than 0.01.

### miRNA-mRNA network construction

**FIGURE 7:**
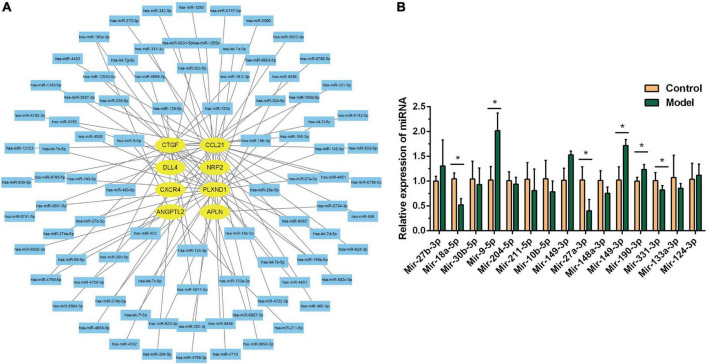
MiRNA-mRNA network construction. **(A)** Interaction network between immune related genes and miRNA. **(B)** Relative miRNA expression in control group and model group. *P < 0.05.

The miRTarBase and miRDB databases were used to predict the miRNAs of core genes. As shown in Figure 7A, 91 miRNAs were screened, which can be verified experimentally. These miRNAs can also be used to treat DHF. We verified 15 miRNAs on animal models by RT-PCR, and the results were shown in Figure 7B, the differential miRNAs were miR-27a-3p, miR-18a-5p, miR-9-5p, miR-190a-3p, miR-149-3p, miR-331-5p. The expression of miR-9-5p, miR-190a-3p, and miR-149-3p were up-regulated, while miR-27a-3p, miR-18a-5p, and miR-331-5p were down-regulated.

### Validation of core genes

**FIGURE 8:**
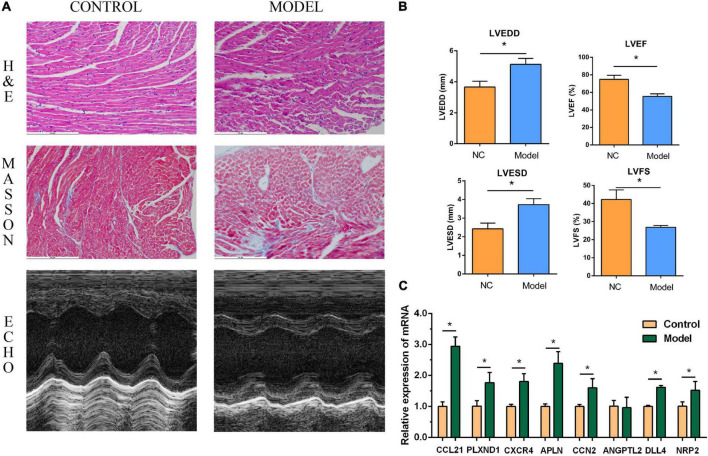
Myocardial injury and impaired heart function were assessed in Type 1 diabetic heart failure mice model. **(A)** Representative pictures of HE staining, Masson staining, and echocardiography (magnification, 400 ×). **(B)** Comparison of LVEDS, LVEDD, LVEF, and LVFS between the control group and the model group. **(C)** Relative mRNA expression of each core gene in control group and model group. *P < 0.05.

**FIGURE 9:**
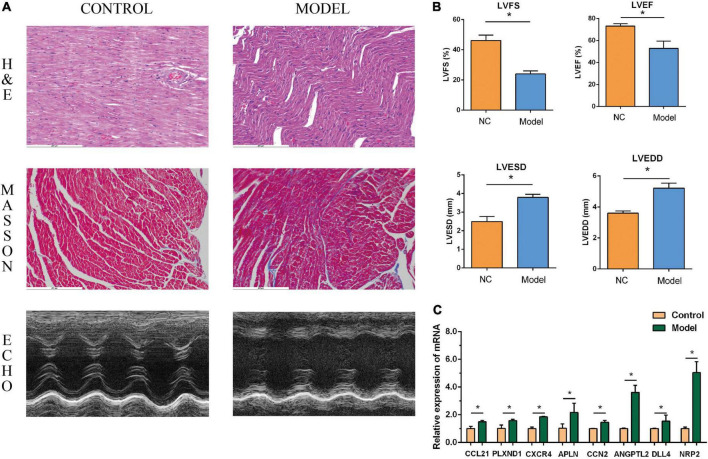
Myocardial injury and impaired heart function were assessed in Type 2 diabetic heart failure mice model. **(A)** Representative pictures of HE staining, Masson staining, and echocardiography (magnification, 400 ×). **(B)** Comparison of LVEDS, LVEDD, LVEF, and LVFS between the control group and the model group. **(C)** Relative mRNA expression of each core gene in control group and model group. *P < 0.05.

The performance of the core genes in the GSE26887 dataset were evaluated. As shown in Figure 4C, all genes had good prediction ability, and all area under the curve (AUC) values were greater than 0.7. Next, the model of DHF mice was established using HE staining, Masson staining, and echocardiography (Figures 8A,B, 9A,B). The mRNA levels of the eight core genes in mouse heart samples were detected using RT-PCR. As shown in Figures 8C, 9C, compared with the control group, core genes were highly expressed in the DHF group.

## Discussion

In recent years, the incidence of diabetes has increased and is accompanied by various complications, especially cardiovascular diseases. A previous study found that the risk of congestive HF in older patients with type 2 diabetes mellitus (DM) was 1.3 times that in those without type 2 DM (15). Therefore, it is necessary to explore the pathogenesis of and biomarkers for DHF. In this study, DEGs between DHF and NHF were screened and DE-IRGs were obtained through intersection with IRGs. In addition, eight core genes were identified in the PPI network. CXCR4, CCN2, DLL4, PLXND1, APLN, NRP2, CCL21, and ANGPTL2 were defined as core genes and verified using *in vivo* experiments. Besides, neutrophils may play an important role in DHF. Finally, the correlation between each core gene and immune cells were analyzed and an miRNA-mRNA network was constructed.

In this study, transcriptome results of diabetic or non-diabetic patients with heart failure in the dataset (GSE26887) were selected. However, we did not select normal heart samples as controls but used heart samples of diabetic or non-diabetic patients with heart failure. Because we found that some inflammatory indicators such as interleukin-6 and interleukin-18 were abnormally elevated in normal heart samples when we processed the data, which was inconsistent with common sense. However, there are few abnormal transcriptome results in the heart of diabetic or non-diabetic patients with failure, so we choose the heart samples of diabetic or non-diabetic patients with failure. In animal models, we verify the selected core genes, and the results are just as we expected.

CXCR4 is a chemokine receptor composed of 352 amino acids (16) and has been proven to be related to the regeneration of many organs and tissues such as the lungs, coronary artery, nervous system, and liver (17, 18). Studies have shown that the expression of CXCR4 increased significantly after acute myocardial infarction in diabetic mice (19). Weng et al. found that fibroblasts can aggravate diabetic cardiomyopathy through the CXCR4/SDF-1 axis (20). CCN2, also called connective tissue growth factor (CTGF), plays an important role in various fibrotic diseases (21, 22). A previous study found that rats with STZ-induced diabetic cardiomyopathy overexpressed CCN2, which could be alleviated by N-acetylcysteine (23). Studies have also shown that CCN2 can be used as a marker for cardiac remodeling in STZ-induced diabetic mice (24). DLL4, a ligand of the Notch receptor, plays an important role in angiogenesis and vascular remodeling. It has also been shown to be a core gene in diabetic vascular injury (25). In addition, a study has shown that the DLL4-NOTCH 1 pathway aggravates vascular permeability in diabetic retinopathy (26). However, no relationship has been found between DLL4 and DHF or cardiomyopathy. This may explain the new mechanism underlying DHF. PLXND1 was initially studied as a tumor promoter and suppressor, but could also play a regulatory role in the development of cardiovascular disease. There is evidence that after knockout of PLXND1, mice will have cardiovascular system injuries, such as abnormal aortic arch and reduced ventricular wall thickness (27). APLN is a G protein-coupled receptor mainly expressed in the nervous and cardiovascular systems. Blood APLN levels of patients with type 2 DM tend to rise (28). A study has also shown that apelin therapy can increase capillary density to improve diabetic cardiomyopathy (29). However, this seems to be contradictory. Therefore, it is necessary to determine the specific role of APLN in DHF. NRP2 is important in angiogenesis, antigen presentation, and phagocytosis, and promotes atherosclerosis (30, 31). However, the effect of NRP2 on diabetic heart injury remains unknown. CCL21 regulates T cells and dendritic cells, and participates in immune responses. Research has shown that CLL21 levels increase after acute myocardial infarction (32) and may play a pathogenic role in HF (33). The results of the present study also showed this, and CLL21 was found to be highly expressed in DHF. This could be a new target for the treatment of DHF. Studies have shown that ANGPTL2 increases in HF, and higher ANGPTL2 levels will increase the risk of HF (34) and ANGPTL2 is an independent risk factor for diabetes development (35). This study is the first to show that ANGPTL2 expression is increased in DHF. The GO and KEGG results of core genes showed that angiogenesis is a common biological process, similar to the above discussion, which indicates that angiogenesis plays an important role in DHF.

Immune cells play an important role in ischemic HF. Neutrophils and macrophages infiltrate the ischemic area (36), an important process in ischemic tissue healing. However, diabetes can cause changes in the number and function of the immune cells. To the best of the knowledge of the authors, this is the first study on immune cell infiltration in DHF. According to the results of the present study, the numbers of activated dendritic cells, central memory CD4 T cells, central memory CD8 T cells, MDSCs, neutrophils, and regulatory T cells were significantly higher in the DHF group than in the control group. Dendritic cells are antigen-presenting cells responsible for consuming antigens and pathogens and producing an MHC-antigen peptide complex. Previous research has found that inflammatory dendritic cells infiltrate the heart tissue of diabetic cardiomyopathy mice and promote the development of an inflammatory environment and disease development (37, 38). In addition, it was found that memory T cells increased significantly in the heart samples from patients with DHF. Some researchers found that, compared with the control group, the memory T cells of diabetic patients increased significantly, and the effect of memory T cells could be used as a potential biomarker for cardiovascular diseases in diabetic patients (39, 40). It has also been suggested that type 2 DM, as a factor contributing to aging (41, 42), leads to the accumulation of end-stage memory T cells. The accumulated aging T cells showed impaired migration function and could harm immune function (43). MDSCs are derived from the bone marrow and are precursors of dendritic cells, macrophages, and granulocytes, which can significantly inhibit the immune response. Although there is evidence that MDSCs are elevated in patients with diabetes (44, 45), it was found for the first time that MDSCs were elevated in cardiac samples of patients with DHF, which also provided a therapeutic direction for myocardial injury caused by diabetes. Interestingly, regulatory T cells were higher in the DNF group than in the control group, based on the results obtained in the present study. Regulatory T cells can inhibit the activation and proliferation of T cells and regulate immunity. Previous evidence has shown that regulatory T cell levels in mice with diabetic cardiomyopathy were significantly reduced, and that regulatory T cells could improve diabetic cardiomyopathy (46). Further studies are needed to confirm the role of regulatory T cells in DHF. As the first line of defense in the inflammatory response, neutrophils play an important role in the immune response. Studies have shown that neutrophil-to-lymphocyte ratios (NLR) are associated with diabetic heart damage (47, 48). A recent study suggested that inflammatory serine proteases produced by neutrophils can aggravate diabetic cardiomyopathy and promote the development of HF (49). In addition, the correlation between core genes and neutrophils were elucidated in this study. The results showed a significant correlation between all core genes and neutrophils, especially CXCR4, ANGPTL2, and DLL4. There is evidence that CXCR4 is associated with neutrophil migration and homing (50); however, the mechanism of DHF is unknown. The core genes and immune cells identified in this study may explain the causes of DHF.

The present study had several advantages. First, the authors are the first to analyze immune cell infiltration in DHF and provide a new approach for treating DHF. Second, the core genes in the animal samples were verified to ensure reliability of the results. Third, an miRNA network related to core genes was constructed, which may provide a new approach for the treatment of diabetic heart injuries.

Some limitations also existed in the present study. First, only one GEO dataset was selected, and there were insufficient samples in the dataset, which may not be conducive to the screening of core genes. Second, the critical value selected for screening the genes was relatively low. Although they have been verified in animal samples, they still need verification in clinical samples. Third, Although we report that immune-related cells are involved in the development of diabetic cardiomyopathy, we have not further verified it in immunofluorescence or immunohistochemistry experiments, and it needs further development and verification in the future, with huge potential. Fourth, although we have verified that there were differences between IRGs and miRNAs in animal models, subsequent experiments are still needed to prove whether there is a causal relationship between genes and miRNAs.

## Conclusion

In conclusion, there are differences in immune infiltration between patients with DHF and NHF. The increase in neutrophils and the expression of the core genes CXCR4, CCN2, DLL4, PLXND1, APLN, NRP2, CCL21, and ANGPTL2 may be highly correlated with DHF. To date, the relationship between core genes and immune infiltration in DHF has rarely been reported. The results of the present study explain the pathogenesis of DHF from the perspective of immune infiltration.

## Data availability statement

The datasets presented in this study can be found in online repositories. The names of the repository/repositories and accession number(s) can be found in the article/Supplementary material.

## Ethics statement

This animal study was reviewed and approved by the Animal Care and Use Committee of Shaoxing People’s Hospital.

## Author contributions

ZZ, TX, and HZ: conceptualization, methodology, design of the research, writing, and original draft preparation. ZZ and SS: bioinformatic data collection and analysis. ZZ, JH, TX, XC, and HL: experimental data collection. JS, JY, XC, and HL: experimental data analysis. ZZ, JH, and HG: software validation and result interpretation. ZZ: preparation of figures. ZZ and HG: review, revision, and editing. All authors approved the final version of the manuscript.

## Footnotes

1
https://www.ncbi.nlm.nih.gov/geo/

2
https://www.immport.org

3
https://cn.string-db.org/

4
http://mirdb.org/

5
https://mirtarbase.cuhk.edu.cn/~miRTarBase/miRTarBase_2022/php/index.php

## Supplementary material

Supplementary Figure 1 GO and KEGG enrichment analysis of DEGs.
