# Supplementary material for: Identification and verification of immune-related biomarkers and immune infiltration in diabetic heart failure
Source: Front Cardiovasc Med. 2022 Nov 17;9:931066. doi: 10.3389/fcvm.2022.931066 (PMC9712450; doi:10.3389/fcvm.2022.931066)
Supplement: Supplementary file 1 [file Table_1.docx]

TABLE S1 Primer sequences of RNAs for qRT-PCR

| Gene |  | Forward primer (5'-3') |  | Reverse primer (5'-3') |
| --- | --- | --- | --- | --- |
| CXCR4 |  | CGTCGTGCACAAGTGGATCT |  | GTTCAGGCAACAGTGGAAGAAG |
| CCN2 |  | GACCCAACTATGATGCGAGCC |  | CCCATCCCACAGGTCTTAGAAC |
| DLL4 |  | TTCCAGGCAACCTTCTCCGA |  | ACTGCCGCTATTCTTGCCCC |
| PLXND1 |  | CGCAACCGTAGCCTAGAAGAC |  | GGTTAAGGTCGAAGGTGAAGAG |
| APLN |  | TGAATCTGAGGCTCTGCGTG |  | ACATCAGTGGCACTCCACAA |
| NRP2 |  | GCTGGCTACATCACTTCCCC |  | GGGCGTAGACAATCCACTCA |
| CCL21 |  | CCCCGGCTGCAGGAA |  | TGTTCAGTTCTCTTGCAGCCC |
| ANGPTL2 |  | CCACCTCGGGTCTACCAAC |  | CTTGCAGGCAGTCTCTCCAT |
| β-action |  | TATAAAACCCGGCGGCGCA |  | TCATCCATGGCGAACTGGTG |
| mmu-mir-27b-3p |  | AACCGGTTCACAGTGGCTAAGTT |  | GTCGTATCCAGTGCAGGGTCC |
| mmu-mir-18a-5p |  | AAGCGGATAAGGTGCATCTAGTG |  | CAGTGCAGGGTCCGAGGT |
| mmu-mir-30b-5p |  | AAGCGCCTTGTAAACATCCTACA |  | CAGTGCAGGGTCCGAGGT |
| mmu-mir-9-5p |  | ACGCCGTCTTTGGTTATCTAGCT |  | CAGTGCAGGGTCCGAGGT |
| mmu-mir-204-5p |  | AACCGGTTCCCTTTGTCATCCTA |  | CAGTGCAGGGTCCGAGGT |
| mmu-miR-211-5p |  | AACCTCCTTCCCTTTGTCATCCT |  | CAGTGCAGGGTCCGAGGT |
| mmu-mir-10b-5p |  | AACAGTGTACCCTGTAGAACCGA |  | CAGTGCAGGGTCCGAGGT |
| mmu-mir-148a-3p |  | AACAATGAGGGAGGGACGGG |  | CAGTGCAGGGTCCGAGGT |
| mmu-mir-27a-3p |  | AACCTCCTTCACAGTGGCTAAGT |  | GTCGTATCCAGTGCAGGGTCC |
| mmu-mir-149-3p |  | AACAATGAGGGAGGGACGGG |  | CAGTGCAGGGTCCGAGGT |
| mmu-mir-190-3p |  | CGCGGCCACTATATATCAAGCAT |  | CAGTGCAGGGTCCGAGGT |
| mmu-mir-331-3p |  | AACAAGCTAGGTATGGTCCCAGG |  | CAGTGCAGGGTCCGAGGT |
| mmu-mir-211-5p |  | AACACGCTTCCCTTTGTCATCC |  | CAGTGCAGGGTCCGAGGT |
| mmu-mir-133a-3p |  | AACAGTGTTTGGTCCCCTTCAAC |  | CAGTGCAGGGTCCGAGGT |
| mmu-mir-124-3p |  | AAGTACTCTAAGGCACGCGGT |  | CAGTGCAGGGTCCGAGGT |
| U6 |  | CTCGCTTCGGCAGCACATATACT |  | ACGCTTCACGAATTTGCGTGTC |
