# Supplementary figures and images for: Identification and verification of immune-related biomarkers and immune infiltration in diabetic heart failure
Source: Front Cardiovasc Med. 2022 Nov 17;9:931066. doi: 10.3389/fcvm.2022.931066 (PMC9712450; doi:10.3389/fcvm.2022.931066)

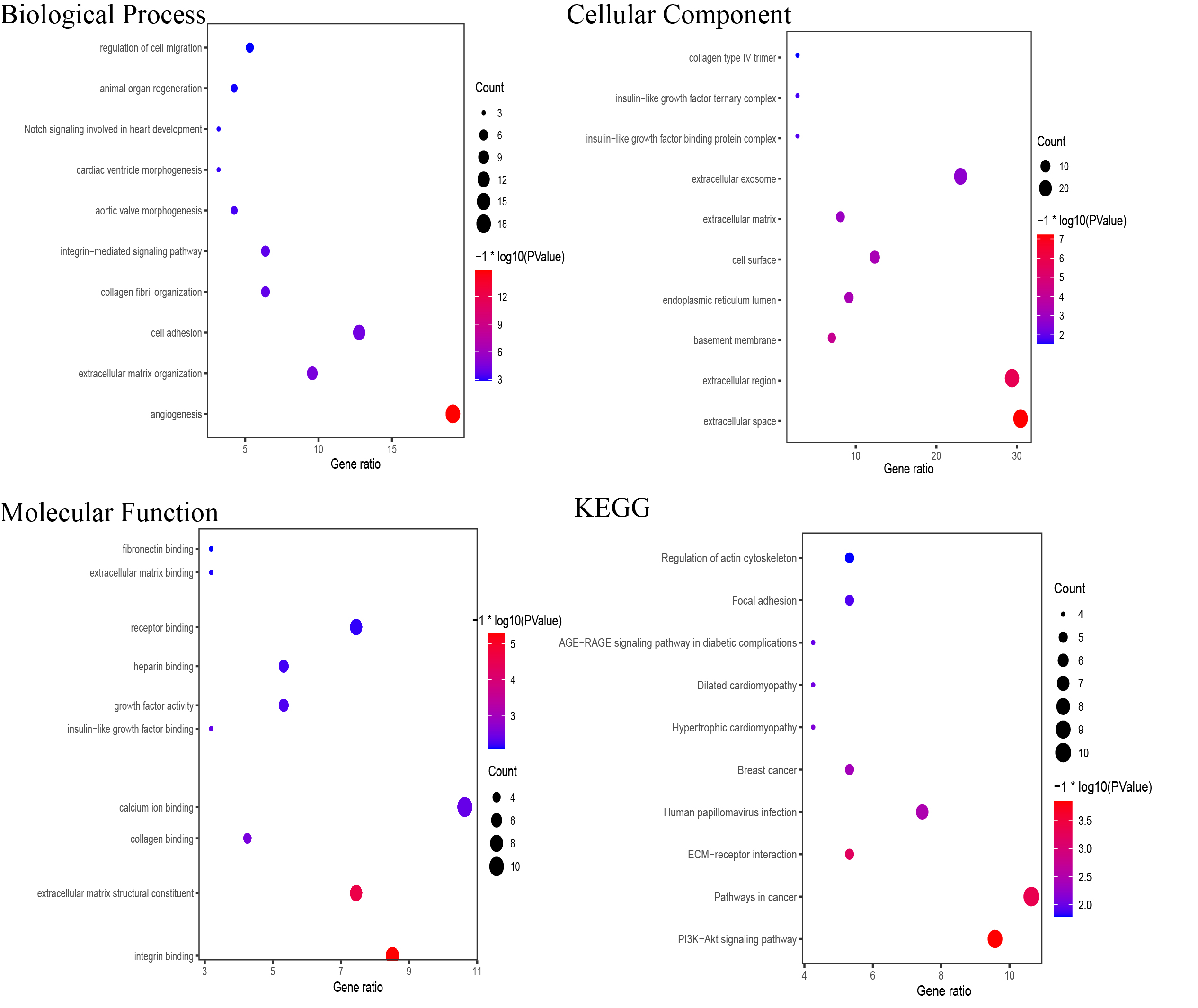

Supplement: Supplementary Figure 1 — GO and KEGG enrichment analysis of DEGs. [file Image_1.JPEG]
